# Association of Hypertension Severity with 30-Day Major Adverse Cardiovascular Events in Patients with Intermediate High-Sensitivity Cardiac Troponin I

**DOI:** 10.5811/westjem.48347

**Published:** 2026-05-14

**Authors:** Kegham Hawatian, Joshua Emakhu, Thayer Morton, Arqam Husain, Hashem Nassereddine, Munir Sidani, Bernard Cook, Howard Klausner, James McCord, Satheesh Gunaga, Seth Krupp, Joseph Miller

**Affiliations:** * Henry Ford Health and Michigan State University Health Sciences, Department of Emergency Medicine, Detroit, Michigan; † Henry Ford Health and Michigan State University, Department of Pathology, Detroit, Michigan; ‡ Henry Ford Health and Michigan State University, Department of Medicine, Division of Cardiology, Detroit, Michigan; § American University of Beirut Medical Center, Department of Internal Medicine, Beirut, Lebanon; || Corewell Health, Department of Emergency Medicine, Royal Oak, Michigan

## Abstract

**Introduction:**

Hypertension is a recognized risk factor for acute coronary syndrome and major adverse cardiovascular events, yet its influence on high sensitivity cardiac troponin (hscTn) concentrations and on the prognostic value of intermediate hscTnI results remains uncertain. We assessed whether blood pressure category confounds the relationship between intermediate hscTnI values (4–18 nanograms per liter [ng/L]) and 30-day major adverse cardiovascular events.

**Methods:**

We performed a secondary analysis of the Rapid Acute Coronary Syndrome Evaluation-Implementation Trial (steppedwedge randomized trial across nine Michigan emergency departments [ED] (July 2020–April 2021). From 32,609 patients in the primary trial, we analyzed only those with available hs-cTnI values reported to be in the intermediate range (4–18 ng/L). The first recorded ED blood pressure-determined category: normotensive (< 140/< 90 millimeters of mercury [mm Hg] moderate [140–179/90–109 mm Hg]; or severe [≥ 180/≥ 110 mm Hg]. Generalized linear models and penalized logistic regression examined associations with hscTnI and 30-day major adverse cardiovascular events (allcause death, myocardial infarction, or urgent revascularization), respectively, adjusting for confounders.

**Results:**

Analysis included 23,803 patients. Mean age was 57.5 ± 17.9 years; 57.5% were women and 32.7% Black. Blood pressure categories were normotensive 40.9%, moderate 46.5%, and severe 12.6%. After adjustment, severe blood pressure was associated with a 16% higher mean hscTnI (calculated as %change= 10^β^ − 1; β = 0.064, 95%, CI 0.047–0.082). Major adverse cardiovascular events at 30-days occurred in 148 patients (0.6%), 47 of them normotensive (0.5%), 77 with moderate hypertension (0.7%), and 24 with severe hypertension (0.8%). Compared with normotension, moderate blood pressure independently increased 30-day major adverse cardiovascular events risk (adjusted odds ratio [AOR] 1.47, 95% CI, 1.02–2.13; absolute risk difference +0.25%, 95% CI, 0.01–0.49), whereas severe blood pressure showed no clear association (AOR 1.32, 95% CI, 0.80–2.18; absolute risk difference +0.17%, 95 % CI, −0.09 to 0.43). Estimates were similar in sensitivity analyses limited to patients without coronary artery disease.

**Conclusion:**

Among ED patients with intermediate hscTnI, blood pressure ≥ 140/90 mm Hg confers modestly higher short term risk of major adverse cardiovascular events, but incremental severity beyond this threshold does not add prognostic value. Elevated hscTnI in the context of severely elevated blood pressure likely reflects myocardial stress rather than additional ischemic risk. Clinicians should interpret intermediate troponin results in hypertensive patients cautiously, integrating clinical presentation and established risk factors.

## INTRODUCTION

Hypertension remains the leading modifiable contributor to global cardiovascular morbidity and mortality.^1^,^2^ In the emergency department (ED), hypertension frequently coexists with chest pain and may both elevate highsensitivity cardiac troponin (hscTnI) through myocardial strain and increase the baseline probability of acute coronary syndrome.^3^–^5^ Contemporary hscTnI assays detect concentrations as low as 2–3 nanograms per liter (ng/L); values < 4–5 ng/L identify patients at very low risk of adverse events, whereas values ≥ 19–20 ng/L are consistent with myocardial injury.^6^–^8^ The intermediate zone (4–18 ng/L), therefore, poses diagnostic uncertainty, particularly when comorbid conditions raise troponin in the absence of type 1 myocardial infarction, such as renal dysfunction, pulmonary embolism, or marked blood pressure elevation.^9^–^11^

Whether the severity of blood pressure elevation modifies the prognostic meaning of intermediate hscTnI is unclear. Prior work linked chronic hypertension to lowgrade troponin release and to longterm risk of heart failure,^12^,^13^ but evidence in the acute care setting is sparse. We hypothesized that higher blood pressure categories would correlate with higher mean hscTnI yet would not independently predict shortterm major adverse cardiovascular events after adjustment for traditional risk factors. Understanding this relation could refine ED risk-stratification algorithms and guide clinical decision-making when faced with uncertain laboratory values. We aimed to determine whether hypertension category influences 30-day major adverse cardiovascular events outcomes in patients with intermediate hs-cTnI levels.

## METHODS

### Study Design and Population

We performed a post-hoc, secondary analysis of the Rapid Acute Coronary Syndrome Evaluation–Implementation Trial (RACE-IT), a stepped-wedge, randomized trial across nine EDs (five academic and four freestanding EDs) that enrolled 32,609 patients evaluated for possible myocardial infarction from July 2020–April 2021 in southeast Michigan. 14 The RACE-IT trial exclusion criteria were < 18 years of age, ST-segment elevation myocardial infarction, hs-cTnI levels > 18 ng/L in the ED, trauma, transfers from another facility, residence outside Michigan, and hospice. For this secondary analysis, we included only patients with available hs-cTnI values. All hs-cTnI were tested on the same immunoassay analyzer, Access (Beckman Coulter Inc, Brea, CA), and patients were included if their troponin values fell within the lowest 99th percentile of hs-cTnI values without them being expressively negative—namely hs-cTnI levels between 4–18 ng/L.

This secondary analysis adhered to the Strengthening the Reporting of Observational Studies in Epidemiology (STROBE) reporting guideline, 15 and the institutional review board approved the study with waiver of informed consent.

**Population Health Research Capsule**

What do we already know about this issue?

*Hypertension is linked to troponin elevation and cardiovascular risk, but its effect on intermediate hs-cTnI prognostic value is unclear*.

What was the research question?

*Does hypertension severity modify 30-day MACE risk in ED patients with intermediate hs-cTnI?*

What was the major finding of the study?

*Moderate HTN is associated with MACE (aOR 1.47, 95% CI 1.02–2.13; P=.04); severe HTN was not significant*.

How does this improve population health?

*Refines ED risk stratification by showing moderate HTN raises risk associated with intermediate hs-cTnI elevations*.

### Hypertension Severity Classification

The first non‐invasive blood pressure recorded at triage (automated oscillometric or calibrated manual) defined blood pressure category. Although systolic blood pressure < 130 millimeters of mercury (mm Hg) is now considered normotensive, for the study period we followed prior guidelines to classify hypertension severity: normotensive (< 140/< 90 mm Hg); moderate (140–179/90–109 mm Hg); or severe (≥ 180/≥ 110 mm Hg). 1

### Outcomes

The primary outcome was logtransformed hscTnI concentration. The secondary outcome was 30day major adverse cardiac event, a composite of allcause death, type 1 or type 2 myocardial infarction (Fourth Universal Definition) and urgent revascularization (percutaneous coronary intervention or coronary artery bypass graft). 18 We ascertained outcomes through electronic health record review and linkage to a statewide health information exchange network.

### Statistical Analysis

Descriptive and baseline characteristics for hypertension severity and its outcomes were summarized as means ± standard deviations (SD) for continuous variables, median (interquartile range [AQR]) for non-normally distributed variables, and frequency (%) for categorical variables. We assessed differences between hypertension classification groups using analysis of variance for continuous variables, Kruskal-Wallis tests for skewed distributions, and chi-square (χ^2^) tests for categorical variables. We used a generalized linear modeling framework to evaluate how hypertension severity relates to mean hs-cTnI values while controlling for confounding factors. Model coefficients (β) were reported to indicate the direction and magnitude of association between hypertension severity and hs-cTnI, providing an interpretable measure of effect size in addition to statistical significance. Prespecified confounders included age, sex, selfidentified race/ethnicity, prior coronary artery disease, and serum creatinine. We confirmed model assumptions through residual diagnostics and applied a log10 transformation to hs-cTnI values because they displayed a skewed distribution.

We examined how hypertension severity affects 30-day major adverse cardiovascular events with multivariable logistic regression models and used normotensive patients as the reference group, accounting for the same pre-specified confounders. The analysis yielded adjusted odds ratio (aOR) along with their 95% CIs. We also performed a sensitivity analysis that excluded patients with known history of coronary artery disease. The study tested interaction terms between hypertension severity and sex, race/ethnicity, and history of coronary artery disease to determine whether they modified any effects. We used Python 3.13 (Python Software Foundation, Wilmington, DE) for all analyses. We defined statistical significance as a two-tailed *P* value < .05.

## RESULTS

### Baseline Characteristics of the Study Population

The study included 23,803 patients who presented with suspected acute coronary syndrome (Table 1). Cohorts based on blood pressure included the following: 9,733 normotensive (40.9%), 11,064 moderate hypertension (46.5%), and 3,006 with severe hypertension (12.6%). The average age was 57.5 years (SD 17.9), 57.5% were female, 32.7% Black, and 53.7% White. The average age, and the proportion of Black patients, increased incrementally in each hypertension group.

The median hs-cTnI levels at presentation was 4.0 ng/L (IQR 4.0–7.0) for both normotensive and moderately hypertensive cohorts, while severely hypertensive patients exhibited a median of 5.0 ng/L (4.0–8.0). Creatinine levels were consistent across groups (median: 0.9 mg/dL [0.7–1.1]) demonstrating comparable renal function. The severely hypertensive cohort experienced higher rates of diabetes mellitus (27.8%), chronic kidney disease (22.3%), and history of coronary artery disease (11.8%) than patients in other cohorts.

### Association Between Hypertension Severity and Highsensitivity Cardiac Troponin

In adjusted analysis (Table 2), severe blood pressure was associated with 16% higher mean hs-cTnI (*P* <.001). This was calculated from the obtained β =0.064 through the formula % Change = 10^β^ − 1 (% Change = 10^0.064^ − 1 = 15.8%). Conversely, moderate blood pressure was 5% lower (*P* < .001) relative to normotension. This was calculated from the obtained β = −0.0239 through the same formula (% Change = 10^−0.0239^ − 1 = −5.3%). Black race, male sex, creatinine, and prior coronary artery disease independently correlated with higher hs-cTnI (Table 2).

### Association Between Hypertension Severity and 30-Day Major Adverse Cardiac Event

Overall, 148 events occurred (37 deaths, 91 myocardial infarctions, and 20 revascularizations). Unadjusted rates of major adverse cardiac event rose from 0.5% (normotensive) to 0.7% (moderate) and 0.8% (severe) based on hypertension classification (*P* = .04). After adjustment (Table 3), moderate blood pressure remained associated with higher major adverse cardiac event (adjusted odds ratio [aOR] 1.47, 95% CI 1.02–2.13), whereas severe blood pressure did not reach statistical significance (aOR 1.32, 0.80–2.18). Sensitivity analyses after excluding coronary artery disease presented similar significances for age, sex, and Black ethnicity; however, significance in moderate severity hypertension was not obtained due to the smaller sample size obtained after patients with coronary artery disease were excluded (Table 4).

## DISCUSSION

Our results expand on previous work showing that persistent or chronic hypertension leads to subtle cardiac injury, as reflected by elevated troponin in otherwise asymptomatic or stable patients.^12^,^13^,^20^ In particular, older studies suggested a linear relationship between systolic blood pressure severity and adverse cardiovascular outcomes,^21^–^23^ but these analyses typically involved broader populations or used conventional (non high-sensitivity) troponin assays. The ability of high-sensitivity troponin testing to rule out myocardial events—especially in the 4–18 ng/L range—raises unique diagnostic and prognostic questions that have not been completely addressed by earlier investigations.

While the association between hypertension and major adverse cardiac events is well-documented,^4^,^26^,^31^ our study provides nuance by focusing on intermediate hs-cTnI levels. For clinicians, this group is often the most challenging to interpret, as patients fall neither into clearly “low risk” (< 4 ng/L) nor “overt myocardial injury” (> 18 ng/L) categories. We observed that severe hypertension was linked to higher troponin concentrations, consistent with literature describing myocardial strain, microvascular compromise, and left ventricular hypertrophy as central pathophysiologic processes driving troponin release.^3^,^5^,^16^,^24^,^25^ However, the elevated hs-cTnI in these severely hypertensive patients did not translate into a statistically significant increase in 30-day major adverse cardiac events risk in our adjusted models. This divergence may reflect differences between “pressure-related myocardial stress” and “ischemic myocardial injury,” as well as the complex interplay of chronic medical therapies in patients with more severe or longstanding hypertension.^28^,^29^ However, this difference between moderate vs severe hypertension in terms of association with 30-day major adverse cardiac events may be a statistical artifact and must be verified by further study.

Interestingly, our study also identified a slight paradox wherein moderately hypertensive patients (140–179/90–109 mm Hg) had slightly lower adjusted troponin levels compared to normotensive patients yet a modestly increased 30-day risk of major adverse cardiac events. These counterintuitive findings may be explained in part by unmeasured factors (eg, the presence of antihypertensive regimens, which may mitigate subclinical myocardial injury (thus lowering troponin) while other risk factors (eg, atherosclerosis burden, diabetes, or smoking) still predispose to acute coronary syndrome or short-term events.^29^–^31^ Prospective studies, designed specifically to incorporate medication details, could clarify whether certain classes of antihypertensives attenuate troponin release or whether other confounders are at play.

### Clinical and Research Implications

From a clinical standpoint, these results suggest that any hypertension (blood pressure ≥ 140/90 mm Hg) in patients evaluated for possible acute coronary syndrome and an intermediate hs-cTnI result deserves attention, given the modest yet measurable increase in major adverse cardiac events. However, clinicians should not assume that blood pressure ≥ 180/110 mm Hg automatically confers an even higher short-term risk of acute coronary syndrome, although the low major adverse cardiac event rate limited our ability to clearly define risk in this severe hypertension cohort. Our findings reinforce the importance of comprehensive risk stratification, incorporating other well-established risk markers, and risk modification, including blood pressure control.

Future research should explore potential mechanistic links between severe hypertension and troponin release, including the roles of arterial stiffness, diastolic dysfunction, and microvascular disease.^26^,^27^ Large prospective cohorts would allow the investigation of whether more aggressive blood pressure-lowering strategies in patients with intermediate hs-cTnI reduce either troponin levels and/or major adverse cardiac events.

## LIMITATIONS

Our study has several limitations. First, although we adjusted for a range of confounders, residual confounding is possible—especially concerning antihypertensive treatment, acute pain, or anxiety in the ED, which might influence both blood pressure and troponin release, as well as any confounders not captured by electronic health records. This is especially important when considering blood pressure control regimens, which constitute an important confounder that should be considered in similar studies in the future. Second, we relied on a single blood pressure measurement at triage. Patients with transiently elevated readings might have been misclassified, while those with chronically elevated blood pressure could have been underestimated.^17^ Third, our outcome of 30-day major adverse cardiac event had relatively few events (148 total), limiting statistical power to detect small differences or interactions.

Fourth, all hs-cTnI assays were performed on the Beckman Coulter platform, which may be generalized to other hs-cTnI assay manufacturers, but the cut-off values may differ.^16^,^19^ Fifth, although the study was conducted across multiple hospitals, they belong to a single health system; thus, external validity beyond similar U.S. community ED settings requires caution. Lastly, we examined only short-term events; whether severe hypertension portends worse outcomes over longer follow-up warrants prospective study. All these factors may influence the precision of our results, and they should be taken into consideration when interpreting the reported outcomes of our study.

## CONCLUSION

In ED patients who present with possible acute coronary syndrome and intermediate hs-cTnI levels, any blood pressure ≥ 140/90 mm Hg may carry a modestly increased short-term risk of major adverse cardiac event, but incremental severity (≥ 180/110 mm Hg) does not significantly heighten that risk after adjusting for other clinical factors. While severe hypertension does appear to raise troponin levels, it may represent myocardial stress rather than acute ischemic injury. Integrating these findings with established risk variables, physicians should interpret borderline troponin values in hypertensive patients carefully, ensuring that blood pressure is addressed promptly but also recognizing that higher blood pressure readings do not necessarily translate into an outsized risk of a major adverse cardiac event.

## Figures and Tables

**Table 1:** Baseline characteristics by hypertension severity in study assessing whether blood pressure category confounds the relationship between intermediate high sensitivity cardiac troponin values and 30-day major adverse cardiovascular events.

	Overall (N = 23,803)	Normotensive (n = 9,733)	Moderately Hypertensive (n = 11,064)	Severely Hypertensive (n = 3,006)	P value
Demographics					
Age, mean (SD)	57.5 (17.9)	55.7 (18.5)	57.8 (17.4)	62.5 (16.1)	.001*
Sex, n (%)					< .001*
Female	13,678 (57.5)	5,648 (58.0)	6,172 (55.8)	1,858 (61.8)	
Male	10,125 (42.5)	4,085 (42.0)	4,892 (44.2)	1,148 (38.2)	
Race/ethnicity, n (%)					< .001*
Black	7,783 (32.7)	3,044 (31.3)	3,634 (32.8)	1,105 (36.8)	
Hispanic	1,025 (4.3)	451 (4.6)	455 (4.1)	119 (4.0)	
Other	2,216 (9.3)	1,025 (10.5)	980 (8.9)	211 (7.0)	
White	12,779 (53.7)	5,213 (53.6)	5,995 (54.2)	1,571 (52.3)	
Laboratory biomarkers, IQR [Q1, Q3]					
Hs-cTnI, ng/L	4.0 [4.0, 7.0]	4.0 [4.0, 7.0]	4.0 [4.0, 7.0]	5.0 [4.0, 8.0]	
Creatinine, mg/dL	0.9 [0.7, 1.1]	0.9 [0.7, 1.1]	0.9 [0.7, 1.1]	0.9 [0.7, 1.1]	
Comorbidities, n (%)					
Atrial fibrillation	1,950 (8.2)	856 (8.8)	834 (7.5)	260 (8.6)	.003*
Peripheral vascular disease	981 (4.1)	394 (4.0)	417 (3.8)	170 (5.7)	< .001*
Diabetes mellitus	5,439 (22.9)	2,061 (21.2)	2,542 (23.0)	836 (27.8)	< .001*
Hypertension	11,206 (47.1)	3,869 (39.8)	5,316 (48.0)	2,021 (67.2)	< .001*
Chronic kidney disease	4,666 (19.6)	2,068 (21.2)	1,929 (17.4)	669 (22.3)	< .001*
Chronic obstructive pulmonary disease	2,798 (11.8)	1,238 (12.7)	1,222 (11.0)	338 (11.2)	< .001*
Heart failure	2,458 (10.3)	1,092 (11.2)	1,022 (9.2)	344 (11.4)	< .001*
Coronary artery disease	2,545 (10.7)	1,062 (10.9)	1,127 (10.2)	356 (11.8)	.02*
Outcome					
30-day MACE(%)	148 (0.6)	47 (0.5)	77 (0.7)	24 (0.8)	.06

* Significant value.

*BP*, blood pressure; *Hs-cTnI*, high-sensitivity cardiac troponin I; *IQR*, interquartile range; *MACE*, major adverse cardiovascular event; ng/L, nanograms per liter; *SD*, standard deviation.

**Table 2:** Adjusted associations between blood pressure category and log10hscTnI in study assessing whether blood pressure category confounds the relationship between intermediate high sensitivity cardiac troponin values and 30-day major adverse cardiovascular events.

	Coefficient (β) and Percentage Change (%)	95% CI	P value
Age (per year)	.0103	.010, 0.011	< .001
Male sex	.1138	0.103, 0.125	< .001
Race/ethnicity			
White	ref		
Hispanic	0.0164	−0.011, 0.044	.24
Black	0.0851	0.073, 0.097	< .001
Other race	−0.0096	−0.029, 0.010	.34
Hypertension severity			
Normotensive	ref		
Moderate	−0.0239 (+15.8%)	−0.036, −0.012	< .001
Severe	0.0645 (−5.3%)	0.047, 0.082	< .001
Creatinine	0.1082	0.102, 0.114	< .001
Coronary artery disease	0.1380	0.120, 0.156	< .001

*ref*, reference.

**Table 3:** Adjusted associations between blood pressure category and 30-day major adverse cardiac event.

Characteristics	Unadjusted model		Adjusted model	
	OR (95% CI)	P value	OR (95% CI)	P value
Age, years			1.02 (1.01–1.03)	< .001
Sex, male n (%)			2.21 (1.57–3.12)	< .001
Race/ethnicity				
White			ref	
Hispanic			0.53 (0.17–1.68)	.28
Black			0.52 (0.34–0.82)	.004
Other race			0.64 (0.32–1.27)	.20
Hypertension severity				
Normotensive	Ref		ref	
Moderate	1.44 (1.00–2.08)	.048	1.47 (1.02–2.13)	.04
Severe	1.66 (1.01–2.72)	.044	1.61 (0.98–2.65)	.06
Coronary artery disease			5.45 (3.84–7.73)	< .001

*OR*, odds ratio; *ref*, reference.

**Table 4:** Sensitivity analysis excluding patients with history of coronary disease in study assessing whether blood pressure category confounds the relationship between intermediate high sensitivity cardiac troponin values and 30-day major adverse cardiovascular events.

Characteristics	Unadjusted model		Adjusted model	
	OR (95% CI)	P value	OR (95% CI)	P value
Age, years			1.03 (1.02–1.04)	< .001
Sex, male n (%)			2.66 (1.89–3.74)	< .001
Race/ethnicity				
White			ref	
Hispanic			0.47 (0.15–1.48)	.20
Black			0.47 (0.30–0.73)	.001
Other race			0.56 (0.28–1.11)	.10
Hypertension severity				
Normotensive	Ref		ref	
Moderate	1.44 (1.00–2.08)	.048	1.41 (0.98–2.02)	.07
Severe	1.66 (1.01–2.72)	.044	1.53 (0.93–2.51)	.09

*OR*, odds ratio; *ref*, reference.
